# Is it possible to overcome issues of external validity in preclinical animal research? Why most animal models are bound to fail

**DOI:** 10.1186/s12967-018-1678-1

**Published:** 2018-11-07

**Authors:** Pandora Pound, Merel Ritskes-Hoitinga

**Affiliations:** 1 Safer Medicines Trust, PO Box 122, Kingsbridge, TQ7 9AX UK; 2 SYRCLE, Department for Health Evidence, Radboud University Medical Center, PO Box 9101, Route 133, 6500 HB Nijmegen, The Netherlands

**Keywords:** External validity, Preclinical animal models, Translation, Human-relevant methods

## Abstract

**Background:**

The pharmaceutical industry is in the midst of a productivity crisis and rates of translation from bench to bedside are dismal. Patients are being let down by the current system of drug discovery; of the several 1000 diseases that affect humans, only a minority have any approved treatments and many of these cause adverse reactions in humans. A predominant reason for the poor rate of translation from bench to bedside is generally held to be the failure of preclinical animal models to predict clinical efficacy and safety. Attempts to explain this failure have focused on problems of internal validity in preclinical animal studies (e.g. poor study design, lack of measures to control bias). However there has been less discussion of another key factor that influences translation, namely the *external* validity of preclinical animal models.

**Review of problems of external validity:**

External validity is the extent to which research findings derived in one setting, population or species can be reliably applied to other settings, populations and species. This paper argues that the reliable translation of findings from animals to humans will only occur if preclinical animal studies are both internally *and* externally valid. We review several key aspects that impact external validity in preclinical animal research, including unrepresentative animal samples, the inability of animal models to mimic the complexity of human conditions, the poor applicability of animal models to clinical settings and animal–human species differences. We suggest that while some problems of external validity can be overcome by improving animal models, the problem of species differences can never be overcome and will always undermine external validity and the reliable translation of preclinical findings to humans.

**Conclusion:**

We conclude that preclinical animal models can never be fully valid due to the uncertainties introduced by species differences. We suggest that even if the next several decades were spent improving the internal and external validity of animal models, the clinical relevance of those models would, in the end, only improve *to some extent*. This is because species differences would continue to make extrapolation from animals to humans unreliable. We suggest that to improve clinical translation and ultimately benefit patients, research should focus instead on human-relevant research methods and technologies.

## Introduction

Few would dispute that the pharmaceutical industry is in the midst of a productivity crisis [1–7] or that rates of translation from bench to bedside are dismal [8–15]. Similarly, few would disagree that patients are being let down by the current system of drug discovery. Evidence suggests that current levels of investment in pharmaceutical drugs are out of proportion to their impact in terms of value for money or population health [16]. There is not a drug for every disease; several thousand diseases affect humans, of which only about 500 are estimated to have any approved treatments [17]. Many of the treatments that do exist cause dangerous and undesirable reactions in humans [18]. This failure of the drug discovery system not only lets down patients but also uses an enormous amount of resources that could likely be better spent [19]. The failure of the current drug discovery model is an issue of global importance for human health. However, the first step towards resolving this issue is to identify what is going wrong.

While many factors contribute to the poor rates of translation from bench to bedside (including flawed clinical trials [20]), a predominant reason is generally held to be the failure of preclinical animal models to predict clinical efficacy [4, 6, 9, 14, 21] and safety [22, 23]. Efficacy and safety issues account for the majority of failures (52% and 24% respectively) at Phases II and III of clinical trials [24]. Attempts to explain these failures have focused on problems of internal validity in preclinical animal studies (i.e. shortcomings in study design, conduct, analysis and reporting), but there has been relatively little discussion of another key factor that influences translation, namely the *external* validity of preclinical animal models [25]. External validity is usually taken to mean the extent to which research findings derived in one setting, population or species can be reliably applied to other settings, populations and species [26]. It is a key criterion for assessing the credibility of scientific research. In the field of preclinical animal research, where the findings derived from animal studies are intended to have relevance in clinical settings, external validity is of the utmost importance. External validity is sometimes referred to as generalisability. Although the two terms are interchangeable, we use the term external validity here because generalisability, despite having a distinct methodological meaning, is often confused with translatability. In fact the two concepts are discrete; external validity/generalisability *contributes* to translatability [26] and in fact, as we shall argue, it is a prerequisite for the translation of findings from animals to humans.

In this paper we argue that the translation of findings from animals to humans can only occur reliably if preclinical animal studies are both internally *and* externally valid. We consider the relationship between internal and external validity before going on to explore several key aspects that impact external validity in preclinical animal research. We suggest that while some problems of external validity are surmountable, the issue of human–animal species differences is not; species differences will always have an impact on external validity and the ability to translate preclinical findings to humans. We explore the implications of this conclusion.

## The relationship between internal and external validity

External validity is distinct from *internal* validity, which refers to the scientific robustness of a study’s design, conduct, analysis and reporting. Systematic review evidence has revealed that preclinical animal studies suffer from serious problems of internal validity, in particular a failure to take measures to prevent bias, such as random allocation to groups and blinded assessment of outcomes [27–29]. If a study does not take such measures then its internal validity is poor and its findings cannot be relied upon. Studies that lack internal validity will always lack external validity [30]. For example, in the field of stroke, animal studies that were ‘unblinded’ overestimated the effect of the intervention by 13% compared with studies that included blinding [31]. In other words, the lack of blinding led to the benefits of the animal studies being overstated. Because the benefits were not real, they could not be applied to other populations and settings. As such, lack of internal validity led to a lack of external validity.

**Fig. 1:**
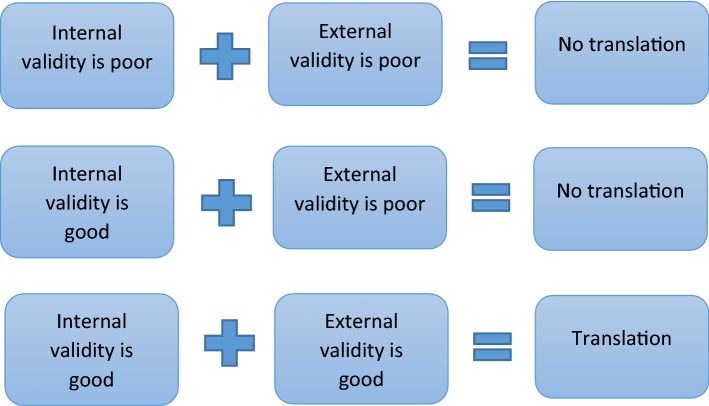
The relationship between internal validity, external validity and translation

Some believe that if issues of internal validity were resolved (i.e. if researchers took measures to prevent bias and conducted studies according to agreed scientific standards) then the clinical translation of animal studies would be more successful [32]. However the available evidence does not support this view. In 1999 the Stroke Treatment Academic Industry Roundtable introduced a series of recommendations and standards intended to improve the quality of animal studies in stroke. Yet by 2012 translation rates had not improved [33] and the situation is no better today. Well over a thousand drugs have been tested in animal studies of stroke [15] but of these only one has translated into clinical use and the benefits of that one are controversial [34]. This may be partly because internal validity remains poor but it is also due to the fact that preclinical animal studies need to be both internally *and* externally valid if they are to translate into benefits for humans (see Fig. 1) [10].

## Aspects of external validity to consider in preclinical animal research

There are several aspects of external validity that raise problems within preclinical animal research. Some of these are potentially surmountable but others are more intractable. We begin this section by reflecting on the surmountable problems of external validity before going on to consider a problem of external validity that we regard as *insurmountable*, namely species differences. We argue that even if the following surmountable issues of external validity were resolved, the issue of species differences would continue to undermine external validity and therefore clinical translation.

### Surmountable problems of external validity

The unrepresentativeness of animal samples is a problem in preclinical research. In general the standardisation of laboratory animal populations produces homogenous samples that do not extrapolate to heterogeneous human populations [26, 35]. In addition, laboratory animals may be housed in conditions that complicate the extrapolation of findings to humans. For example the biology of laboratory mice may be affected by their being housed in same-sex groups, by lack of opportunities for physical exercise, and by temperature [36] and diet [37]. Furthermore, the animals used in preclinical research tend to be young and healthy whereas many human diseases manifest in older age. For example, animal studies of osteoarthritis (OA) tend to use young animals of normal weight, whereas clinical trials focus mainly on older people with obesity [38]. Animals used in stroke studies have tended to be young whereas human stroke is largely a disease of the elderly [39]. It is not hard to see that in such cases the findings from animal studies are unlikely to be applicable to human patient populations, i.e. they will lack external validity.

Furthermore, many animal models lack the complexity required to accurately mimic human conditions. Although there has been some success with diseases based on single gene defects that can be reproduced in animal models [16], most human diseases tend to evolve over time as part of the human life course. For example, it may be possible to grow a breast tumour on a mouse model but this does not actually represent the human experience because most human breast cancer occurs post-menopausally. While some animal species may be better models of specific diseases than others (e.g. horses have similar cartilage degenerative processes as humans [40]), in general the animal models currently used do not mimic the slow, progressive and degenerative nature of many human chronic diseases [10], nor do they involve the complexity of comorbidity or polypharmacy (human patients often take more than one type of medication). To take the example of stroke again, many people with this disease have hypertension but preclinical animal studies of stroke have generally used healthy animals without comorbidities, which results in the effects of interventions being overestimated [31]. In fact many experimental treatments for stroke are less effective in humans with hypertension [39]. Furthermore, recovery from a severe stroke can take years for humans but animals can recover from experimental stroke within days or weeks [41]. Additionally, while human stroke is highly heterogeneous the four most commonly used stroke animal models are all of ischaemic stroke [41].

Finally, animal models developed in the laboratory lack applicability to ‘real life’ clinical settings. To return to the example of OA, animals are usually given drugs for OA prophylactically, or in the early stages of OA, whereas in clinical trials humans are usually given drugs in the late stages of their disease [38]. Similarly, experimental drugs for multiple sclerosis (MS) are most commonly administered to animals some days before neurological impairment. As these drugs may work by blocking the induction of the disease they are not relevant to the human condition because human patients cannot be identified prior to the onset of their MS. Animal models of MS can only have clinical relevance if treatment is successfully started *after* the onset of symptoms [42]. Animal models of Parkinson’s disease pose a similar problem [43], as do those of inflammatory bowel disease [44]. Again, in the case of stroke, Tirilazad was able to successfully treat animals if given within 10 min of stroke induction but humans are highly unlikely to be able to access treatment for stroke within 10 min. In clinical trials humans were given Tirilazad within a more realistic 5 h and the trials were unsuccessful [39]. The choice of animal models across a range of fields appears to lack rationality in terms of evidence-base or appropriateness in relation to the relevant human condition [40, 44].

These failures of animal models to accurately represent human diseases and clinical contexts are sometimes described as failures of construct validity, which is generally understood to be a subset of external validity [10, 45]. As noted above, these failures, which may be in part due to academic pressures to produce quick, high impact papers [46] can potentially be resolved, although some of the solutions raise serious ethical issues (e.g. animal models that involve ageing and comorbidity are likely to be more externally valid but because the harms to animals are protracted they are more likely to be considered severe). Yet even if all the surmountable problems of external validity described above were resolved, one intractable problem would remain. Species differences, i.e. the differences between animals and humans in terms of their underlying biology, would continue to undermine external validity.

### The insurmountable problem of external validity: species differences

Perlman [36, 47], an evolutionary biologist, points out that mice (the most frequently used animals in research) and humans have a high level of genetic homology as well as many biochemical and physiological similarities. He notes however, that the lineages that led to modern rodents and primates diverged around 85 million years ago and that since then, the species in these lineages have become adapted to very different environments. As a result, mice and humans now have very different life histories, they eat different diets, have different levels of physical activity, are exposed to different environmental toxins and pathogens and have different microbiomes. Because they harbour different sets of pathogens and microbiomes, host–pathogen and host–microbiome coevolution has led to evolved differences between the human and mouse immune systems [36, 47]. Furthermore, Perlman [36] suggests that due to different network architectures between mice and humans and different genotype–phenotype relationships, the relationships between genotype and disease are likely to differ in these two species. Critically Perlman notes that while mice and other animals may be useful for understanding processes that arose early in evolution and that humans share with other species, they are less likely to be useful for understanding chronic non-communicable diseases because the pathogenesis of these diseases is enmeshed in our unique, evolved life histories [36, 47, 48].

Nevertheless there is a strong assumption among the biomedical community that gene functions and developmental systems are conserved between animals and humans [49]. Moreover, there appears to be little interest within the biomedical community in verifying this assumption, or in the evidence emerging from evolutionary developmental biology indicating that gene functions and gene networks diverge through evolution [50]. Commentators have observed that the animal model paradigm tends to discourage any critical appraisal of species differences, encouraging instead a view that animal based findings are generally applicable to humans [50, 51] and emphasising the commonalities rather than the differences [21]. Preuss [50] suggests that if species differences are acknowledged they tend to be ‘soft-peddled’ or treated as ‘noise’, again noting that researchers focus on ‘commonalities’ and ‘basic uniformity’ instead. But as Perlman [36] notes, biology is characterized by diversity as well as unity; evolution is ‘descent with modification’ [52].

Unfortunately, focusing on the commonalities without acknowledging difference is problematic. Sjoberg [53] argues that crude inferences are made about the properties of one group (humans) based on observations from another group (animals), simply because both groups have some other property in common (genetic similarity). Sjoberg uses the example of Jack and Jill: if Jack is clumsy then it might be inferred that his sibling Jill is also clumsy. However there is no evidence that Jill is clumsy and the argument is based solely on the observation that Jack and Jill have genetic properties in common. This reasoning, which relies purely on an *assumption* of similarity (rather than its empirical demonstration), underpins the use of animals as models of human disease. As Wall and Shani [21] note, the assumption is that if two systems are homologous then they are likely to function similarly. However this is incorrect; while some molecular pathways may appear identical between humans and animals, there may be differences, for example in specific receptors and enzymes, that will cause them to behave very differently [54]. Non-human primates are often cited as having great genetic similarity with humans, but this belies the fact that in complex living systems even minor differences can result in significant differences in biological processes and outcomes [55]. The case of TGN1412, which was tested in non-human primates precisely because of their close relation with humans, amply demonstrates this. After just a few minutes of being infused with a dose 500 times smaller than that found safe in animal studies, all six human volunteers started suffering severe cytokine release syndrome leading to severe inflammation and multiple organ failure [56]. Wall and Shani [21] suggest that in some cases animal models can serve as a good analogue to study general principles, *but not specific details.* Details matter when it comes to developing safe and effective drugs for humans. As they write, ‘On average, the extrapolated results from studies using tens of millions of animals fail to accurately predict human responses.’ Consequently, they conclude that it is probably inadvisable to use animal models for extrapolation.

## What can be done about the problem of species differences?

Transgenic mouse models were intended to enhance the external validity of animal models but as Geerts [9] suggests, if translation rates are anything to go by they have failed. This is because the paradigm suffers from the same problems; the SOD1 transgenic mouse, for example, appears to mimic humans in terms of some of the characteristics of motor neurone disease, but this is no guarantee that the same mechanisms are involved [10, 57]. Lynch [49] suggests that a way forward might be to empirically demonstrate (rather than assume) the similarity between animal and human genes with regard to the function being studied. Likewise, Seok et al. [12] suggest that researchers should specify in advance the extent to which their animal model mimics the molecular behaviour of the key genes and key pathways thought to be important for the human disease under investigation. While this would appear to provide an answer however, it potentially leads us towards another problem of reasoning, namely the ‘extrapolator’s circle’. In other words, if we want to determine whether a mechanism in animals is sufficiently similar to the mechanism in humans to justify extrapolation, we must know how the relevant mechanism in humans operates. But if we already know about the mechanism in humans then the initial animal study is likely to have been redundant [58] (depending upon the purpose of that animal study [59]).

Consequently we suggest that animal–human species differences constitute a problem of external validity that cannot be overcome. Imagine that the next several decades are spent resolving the myriad problems of internal validity and the surmountable aspects of external validity (i.e. the representativeness of animal samples and the clinical relevance of animal models). While vast resources would be expended and colossal numbers of animals used and killed in this endeavour the end result would be only modest; the robustness of animal studies and the clinical relevance of animal models would likely improve *to some extent*. This unremarkable result would be due to the fact that despite improvements in animal models, the intractable issue of species differences would remain and would continue to make extrapolation from animals to humans unreliable. Along the way there might be some serendipitous findings but serendipity is not a reliable scientific method. Thus decades from now preclinical animal studies would still fail to reliably and consistently predict human responses and the findings from preclinical animal models would still fail to translate into benefits for humans. This is essentially the uninspiring scenario proposed by those who insist that the answer to the problem of translation lies in improving animal studies and animal models. This scenario is particularly unexciting given that current attempts to improve matters have so far failed [33, 60, 61].

An alternative approach, and one taken by an increasing number of scientists, is to consider a paradigm for drug discovery that cuts out the uncertainty introduced by species differences [4, 6, 7, 62]. Within this paradigm new, human-relevant approaches and technologies are considered, such as the generation of human induced pluripotent stem cells (iPSC), which can be used to create disease- or patient-specific cell lines for testing potential drugs, micro-physiological systems known as ‘organs-on-chips’, and human organoids (three dimensional cell cultures that incorporate key features of organs). Many of these new techniques integrate with in silico approaches and with systems biology, seen by many as having potential to revolutionise medicine [63, 64] and drug discovery [2]. Given that the return on investment for developing a new drug decreased from 10% in 2010 to 3% in 2017, the pharmaceutical industry certainly perceives a need to do things differently [5] and there is some considerable optimism, both within industry [4, 6] and elsewhere, about the potential of these new approaches to increase the speed and accuracy of drug discovery. The US is making significant investments in organ-on-chip technologies [7] and the Netherlands is aiming to phase out animal use in the regulatory safety testing of medicines and chemicals by 2025 [65] regarding new technologies as able to increase research relevance and deliver more reliable risk assessments whilst maintaining existing safety levels [66]. Systematic reviews are being used to review legacy data on drug safety [67] and this evidence, alongside low-risk approaches such as microdosing in clinical trials [68], could provide a valuable safeguard during a transition to new technologies [69]. Systematic reviews will also have a role in synthesising emerging evidence about the efficacy of new technologies. Initial findings suggest that organs-on-chips [70, 71] and in silico approaches [72] may have advantages over animal studies in terms of predicting adverse drug reactions. New physiologically relevant technologies also appear more capable of illuminating mechanisms of toxicity than animal studies [73–75].

## Conclusion

We have argued that translation from animals to humans can only occur if preclinical animal studies are both internally and externally valid. We have also suggested that external validity consists of potentially modifiable features (e.g. representativeness of animal samples, clinical relevance of animal models) and unmodifiable features (animal–human species differences). Thus we suggest that while some aspects of animal models can be improved to a limited extent, they can never be fully externally valid because of the uncertainty introduced by animal–human species differences. If the aim is to improve clinical translation and ultimately address patients’ needs for safe and effective treatments, the first step is to acknowledge where current systems are failing.

We noted that those conducting preclinical animal research appear to downplay the problem of animal–human species differences but interestingly, other researchers and commentators in the field do similarly. Although they may briefly acknowledge that species differences constitute a problem for external validity, the tendency is to focus on other, potentially modifiable, aspects of external validity [10, 26]. This is perhaps understandable, since acknowledging the issue of species differences entails confronting the possibility that the preclinical animal research paradigm no longer has a great deal to offer. That possibility is alarming, not only to scientists who conduct animal research but also to those attempting to improve it. Yet there is a way forward. Research methods and technologies that are physiologically relevant to humans obviate the need for animals and thus eliminate the problem of animal–human species differences. As a recent industry report [6] concluded, the time has come to humanise medicine. For the sake of patients and animals, we agree.
